# Sex-specific differences in neuromuscular activation of the knee stabilizing muscles in adults - a systematic review

**DOI:** 10.1186/s40945-022-00158-x

**Published:** 2023-02-15

**Authors:** Martina Steiner, Heiner Baur, Angela Blasimann

**Affiliations:** 1 Division of Physiotherapy, Bern University of Applied Sciences, School of Health Professions, Bern (CH), Switzerland; 2 Faculty of Medicine and Health Sciences, Department of Rehabilitation Sciences and Physiotherapy, University of Antwerp, Wilrijk (BE), Belgium

**Keywords:** Electromyography, EMG, Sex-specific, Sex differences, Females, Males, Adults, Lower extremity, Neuromuscular activation, Healthy

## Abstract

**Introduction:**

The rupture of the anterior cruciate ligament (ACL) is one of the most common injuries of the knee. Women have a higher injury rate for ACL ruptures than men. Various indicators for this sex-specific difference are controversially discussed.

**Aim:**

A systematic review of the literature that compares surface electromyography (EMG) values of adult female and male subjects to find out if there is a difference in neuromuscular activation of the knee stabilizing muscles.

**Methods:**

This systematic review has been guided and informed by the Preferred Reporting Items for Systematic Reviews and Meta-Analysis (PRISMA) guidelines. Studies which examined sex-specific differences with surface EMG measurements (integral, root mean squares, mean values, analysis of time and amplitude) of the knee stabilizing muscles were retrieved via searches from the databases PubMed, CINAHL, Embase, CENTRAL and SPORTDiscus. The risk of bias of included studies was assessed with the National Heart, Lung and Blood Institute (NHLBI) study quality assessment tool. A synthesis of results was performed for relevant outcomes.

**Results:**

Fifteen studies with 462 healthy participants, 233 women (mean age 21.9 (± 2.29) years) and 299 men (mean age 22.6 (± 2.43) years), were included in the systematic review. The methodological quality of the studies was mostly rated “fair” (40%). A significantly higher activity of the muscles vastus lateralis and vastus medialis was found in females, in three studies. Two studies found significantly lower neuromuscular activity in the muscles biceps femoris and semitendinosus in females. All other included studies found no significant differences or reported even contradicting results.

**Conclusion:**

The controversial findings do not allow for a concluding answer to the question of a sex-specific neuromuscular activation. Further research with higher statistical power and a more homogeneous methodical procedure (tasks and data normalisation) of the included studies may provide insight into possibly existing sex-specific differences in neuromuscular activation. This systematic review could help to improve the methodical design of future studies to get a more valid conclusion of the issue.

**Trial registration:**

CRD42020189504.

**Supplementary Information:**

The online version contains supplementary material available at 10.1186/s40945-022-00158-x.



## Introduction

The rupture of the anterior cruciate ligament (ACL) is one of the most common injuries of the knee [1, 2]. Consequences can be severe, such as pain, reduced range of motion, reduced physical activity and long-term joint degeneration [3, 4]. Furthermore, the return to sport rate differs depending on the level of competition: i.e. 81% returned to any sport, 65% returned to the preinjury level of activity and only 55% returned to competition level after surgery [5]. However, after one year only a minority of athletes have returned to their preinjury level [6] and the rate of sustaining a second ACL injury exceeds 20% [7, 8].

Women have a higher injury rate for ACL ruptures than men [9, 10]. This applies in particular for non-contact injuries where women have a three and a half times higher risk to rupture the ACL [11–13]. Another study stated that women playing sports such as soccer or basketball even have a two to eight times higher risk of an ACL rupture [14].

To summarize the points mentioned above, it is important to find out why there is such a large sex difference in non-contact ACL injuries. Various indicators for this increased risk of injury in women, such as biomechanical, hormonal and neuromuscular aspects, are controversially discussed. These contributing factors can be classified into intrinsic (not controllable), extrinsic (controllable), or both (partially controllable) [15]. One study considered the neuromuscular factors as the most likely ones for the increased risk of injury in women [16]. Therefore, the focus of this systematic review will be on neuromuscular factors assessed by EMG, and the comparison of neuromuscular activation between women and men. A narrative review of various cross-sectional studies assumes that the sex difference in injury rates is due to sex-specific neuromuscular adaptation and biomechanical landing techniques [17]. Other studies describe a difference between women and men in activation timing or force intensity of the knee stabilizing muscles [18–23]. Furthermore, a dominance of the quadriceps muscle over the hamstring muscles in women is described. This larger quadriceps to hamstrings ratio could be a risk factor for ACL injuries, as it could promote anterior tibial translation [21, 22, 24, 25]. The quadriceps dominance in women was found in various activities. For example, during jumps, cutting and swerving manoeuvres [26–28]. However, these statements are contradicted by a systematic review which summarizes the sex differences in landing and cutting manoeuvres [29]. A total of seven studies, all cross-sectionally designed, were included in the analysis. The authors summarize that the biomechanical sex differences are based on questionable clinical relevance and that they did not find any quadriceps dominance for the activities described [29]. Furthermore, other authors could not find any sex-specific differences in the explosive quadriceps-hamstrings ratio [30]. In addition, a recent study paired women and men in terms of strength and concluded that strength-paired women and men showed no significant differences in neuromuscular activity [31]. Regarding the controversy of sex-specific differences in neuromuscular activation, it is of general interest to compile data to better understand whether there are sex-specific differences in neuromuscular activation present or not.

The purpose of this systematic review is to find out if there is a difference in neuromuscular activity in adult women and men assessed with electromyography. Results could help as a guideline to the omnipresent question in clinical studies regarding the need to differentiate between women and men in the evaluation of data on neuromuscular activation. Moreover, results could have an impact on the rehabilitation process as the results could focus on a new aspect to consider when defining the rehabilitation strategy for women and men. Consequently, these objectives result in the following research question: Are there sex-specific differences in neuromuscular activation of the knee stabilizing muscles in adults with or without an ACL injury?

## Methods

### Protocol and registration

This systematic review has been guided and informed by the Preferred Reporting Items for Systematic Reviews and Meta-Analysis (PRISMA) guidelines [32, 33]. The protocol has been registered a priori on the International Prospective Register of Systematic Reviews Database – PROSPERO (CRD42020189504).

### Eligibility criteria

To define relevant keywords for the systematic literature search, the Population-Exposure-Comparator-Outcome framework (PECO) was used [34] (Table 1). In addition, the following inclusion criteria were defined: Study participants had to be healthy or suffer from an ACL injury (either treated conservatively or surgically with an ACL reconstruction), comparisons of neuromuscular control between females and males had to be provided, and the EMG-related outcomes had to be reported as mean, root mean squares (RMS), integral, in the domains of amplitude or time etc. Moreover, included studies had to be original articles with any experimental study design (e.g. cross-sectional study, randomized-controlled trial etc.) published in peer-reviewed, scientific journals, and available as full text. No language restrictions were defined. Exclusion criteria were children and adolescents as participants, other injuries of the lower extremity than ACL injuries, neurological diseases, chronic pain or inflammatory processes, data from interventions with a fatigue protocol, and publications such as editorials, book chapters and reviews.

**Table 1:** Overview of PECO criteria [34]

Item	Criteria
Population	Among adults (≥ 18 years of age), what is the effect of
Exposure	neuromuscular activation of the lower limb in females versus
Comparator	neuromuscular activation of the lower limb in males on
Outcomes	parameters describing surface EMG outcomes

Legend: *EMG* electromyography

### Information sources and search strategy

The search was conducted between August and September 2020 using five electronic databases: PubMed, CINAHL, Embase, Cochrane Central Register of Controlled Trials (CENTRAL) and SPORTDiscus. The search strategy was based on the predetermined research question and the PECO method. The following search string was used: “(EMG OR electromyography) AND (lower extremity OR knee OR anterior cruciate ligament OR ACL) AND (neuromuscular activation OR neuromuscular control OR neuromuscular activity) AND (gender bias OR sex differences OR sex factors OR female OR females OR woman OR women OR male OR males OR man OR men)”. The adaption of the search string for each database was conducted with the help of the “Ref-Hunter Manual” for literature research [35]. The database search was completed by checking the reference lists of included studies to identify additional and potentially eligible studies. There was no search for grey literature.

### Study selection

The acquired data was extracted and organized with the Endnote software (EndNote X9.2 (Bld 13,018), Clarivate Analytics, Philadelphia, USA). After exclusion of all duplicates, two reviewers (MS and AB) independently assessed titles and abstracts of the retrieved, eligible literature based on the defined criteria in Table 1. Clearly ineligible studies were excluded. The assessment of the references was coordinated with the Rayyan QCRI software [36]. Disagreements were discussed and resolved by consensus, where necessary, with a third reviewer (HB). Afterwards, a second screening procedure followed. Therefore, all eligible articles were retrieved in full text and reviewed independently for inclusion by the same two reviewers (MS and AB).

### Assessment of risk of bias

The quality of each study included was evaluated using the “Quality Assessment Tool for Observational Cohort and Cross-Sectional Studies” (QAT) from the NHLBI [37]. This assessment tool is widely used and recommended. It includes fourteen questions for evaluating potential bias in studies such as patient selection, study power, confounding and the strength of causality in the association between interventions and outcomes. As a reviewer you can select “yes”, “no” or “cannot determine / not applicable / not reported”. There is no quantitative rating of the level of evidence. After a calibration regarding the assessment tool, both reviewers (MS and AB) assessed the quality of the included studies independently. Disagreements were discussed and solved by consensus. All studies remained included regardless the outcome of the quality assessment.

### Data items, collection process and synthesis methods

Concerning the data items and collection, a spreadsheet was developed a priori to the data extraction. The data extraction was conducted by the first author (MS) followed by an accuracy examination of the last author as second reviewer (AB) to assure quality control. The following variables regarding study characteristics were extracted: first author, publication year, country where study had been conducted, study design, number of participants measured, mean age of participants, intervention/task, outcomes, main results, and conclusion. Quantities of surface electromyography (EMG) measurements such as root mean squares (RMS), integrals, mean values, and analysis in time and amplitude domain were extracted as outcomes.

The weighted overall effect size of the standardized mean difference and the 95% confidence interval (95%-CI) were calculated for each included study. The Hedges’g within a random-effect model was used as a standardization. The software RevMan 5.3 (Review Manager, Version 5.3. Copenhagen: The Nordic Cochrane Centre, The Cochrane Collaboration, 2014) was used. Due to heterogeneity between the studies (e.g. various tasks, different measurement units, different ways to normalize EMG data, various levels of sport of the participants) it was not appropriate to pool the data for the purpose of a meta-analysis. For this reason, a systematic review with a synthesis according to PRISMA [33, 34] was carried out (Additional file 1).

## Results

### Study selection

**Fig. 1:**
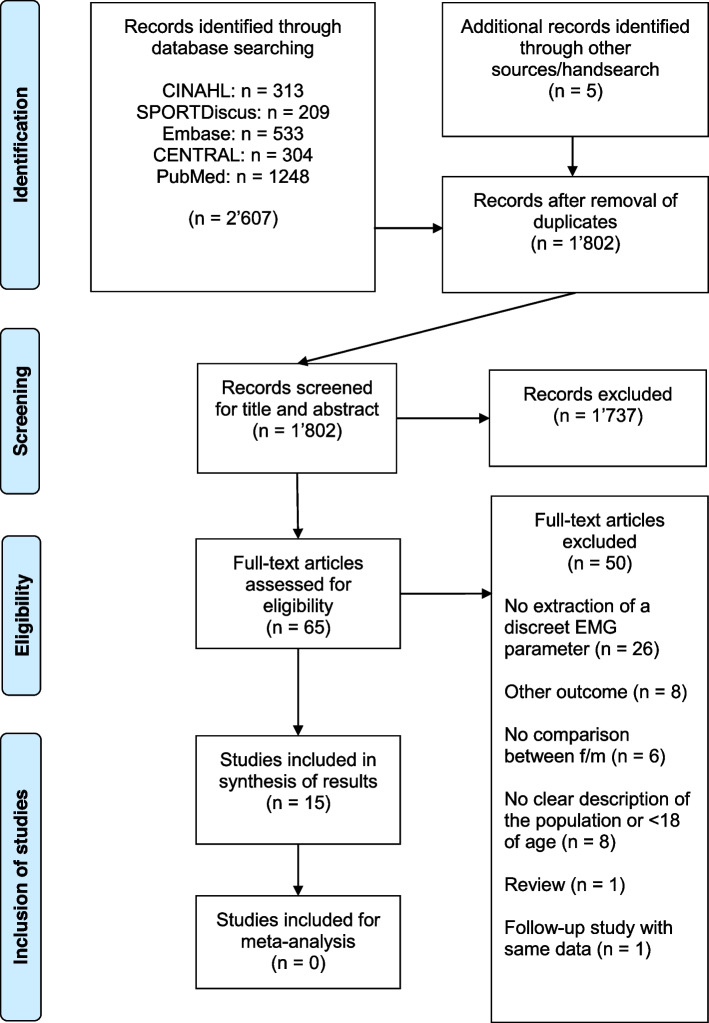
Flow chart of the search strategy, adapted from PRISMA [33]. Abbreviation: f/m = female/male

Figure 1 presents all the screened and included studies in this review. Fifteen studies [20, 22, 24, 27, 28, 38–47] of a total of 2′607 papers retrieved from the database search, met the inclusion criteria.

### Quality assessment

The methodological quality of the 15 included studies was rated according to the NHLBI assessment tool [37] (see Table 2). All studies were cross-sectionally designed. Six studies were rated to have a fair quality (40%), four showed a good quality (27%) and five had a poor quality (33%). The total agreement (good/fair/poor) between the two reviewers amounted to 73% (11/15 studies) and the inter-rater reliability, measured using the Cohen’s Kappa, was good (*K* = 0.6) [48]. The main limitations were (i) no mention of a sample size justification as 11 of 15 studies reported no power description [20, 22, 24, 27, 28, 39–41, 44–46] and (ii) the lack of consideration of confounders as six out of 15 studies had a more heterogeneous subject group without any statistical adjustment [27, 39, 42, 43, 46, 47]. In addition, none of the studies reported blinding of the assessors. Common strengths were the clearly stated objectives and accurate description of the independent and dependent variables, respectively.

**Table 2:** Quality assessment scores according to the NHLBI quality assessment tool for observational cohort and cross-sectional studies [37]

Study (publication year)	Q1	Q2	Q3	Q4	Q5	Q6	Q7	Q8	Q9	Q10	Q11	Q12	Q13	Q14	Quality Rating
Bencke & Zebis (2011) [38]	Yes	Yes	Yes	NR	Yes	No	No	NA	Yes	NA	Yes	NR	NA	Yes	good
DeMont & Lephart (2004) [39]	Yes	Yes	Yes	NR	No	No	No	NA	Yes	NA	Yes	NR	NA	No	poor
Deschenes et al. (2009) [40]	Yes	Yes	Yes	NR	No	Yes	Yes	No	Yes	NA	Yes	NR	Yes	Yes	fair
Hanson et al. (2008) [24]	Yes	Yes	Yes	Yes	No	No	No	NA	Yes	NA	Yes	NR	NA	Yes	fair
Hart et al. (2007) [41]	Yes	Yes	Yes	Yes	No	No	No	NA	Yes	NA	Yes	NR	NA	Yes	good
Kim et al. (2016) [42]	Yes	Yes	Yes	Yes	Yes	No	No	NA	Yes	NA	Yes	NR	NA	No	fair
Lee et al. (2015) [43]	Yes	Yes	Yes	NR	Yes	No	No	NA	Yes	NA	Yes	NR	NA	No	poor
Myer et al. (2005) [27]	Yes	Yes	Yes	Yes	No	No	No	NA	Yes	NA	Yes	NR	NA	No	fair
Padua et al. (2005) [28]	No	Yes	Yes	Yes	No	No	No	NA	Yes	NA	Yes	NR	NA	Yes	poor
Palmieri-Smith et al. (2007) [44]	Yes	Yes	Yes	Yes	No	No	No	NA	Yes	NA	Yes	NR	NA	Yes	fair
Rozzi et al. (1999) [45]	Yes	Yes	Yes	Yes	No	No	No	NA	Yes	NA	Yes	NR	NA	Yes	good
Shultz et al. (2001) [20]	Yes	Yes	Yes	NR	No	No	No	NA	Yes	NA	Yes	NR	NA	Yes	good
Smith et al. (2009) [46]	Yes	No	Yes	Yes	No	No	Yes	NA	Yes	NA	Yes	NR	NA	No	poor
Urabe et al. (2004) [22]	Yes	Yes	Yes	Yes	No	No	No	NA	Yes	NA	Yes	NR	NA	Yes	fair
Wu et al. (2016) [47]	Yes	Yes	Yes	NR	Yes	No	No	Yes	Yes	NA	Yes	NR	NA	No	poor

Legend: *NHLBI* National Heart, Lung, and Blood Institute, *NA* not applicable, *NR* not reported, *Q* Question, Q1 = Was the research question or objective in this paper clearly stated?; Q2 = Was the study population clearly specified and defined?; Q3 = Was the participation rate of eligible persons at least 50%?; Q4 = Were all the subjects selected or recruited from the same or similar populations (including the same time period)? Were inclusion and exclusion criteria for being in the study prespecified and applied uniformly to all participants?; Q5 = Was a sample size justification, power description, or variance and effect estimates provided?; Q6 = For the analyses in this paper, were the exposure(s) of interest measured prior to the outcome(s) being measured?; Q7 = Was the timeframe sufficient so that one could reasonably expect to see an association between exposure and outcome if it existed?; Q8 = For exposures that can vary in amount or level, did the study examine different levels of the exposure as related to the outcome (e.g., categories of exposure, or exposure measured as continuous variable)?; Q9 = Were the exposure measures (independent variables) clearly defined, valid, reliable, and implemented consistently across all study participants?; Q10 = Was the exposure(s) assessed more than once over time?; Q11 = Were the outcome measures (dependent variables) clearly defined, valid, reliable, and implemented consistently across all study participants?; Q12 = Were the outcome assessors blinded to the exposure status of participants?; Q13 = Was loss to follow-up after baseline 20% or less?; Q14 = Were key potential confounding variables measured and adjusted statistically for their impact on the relationship between exposure(s) and outcome(s)?

### Study participants

In total, 462 healthy participants, 233 women and 229 men, were tested. The mean age was 21.9 (± 2.29) years for women and 22.6 (± 2.43) years for men. Excluded from this calculation are 20 participants from the study of Wu et al. (2016) [47]. In this study an older group with a mean age of 66.8 ± 3.4 years for men and 66.1 ± 4.4 years for women had been measured (see Table 3).

**Table 3:** Characteristics of the included studies

**Study (publication year) Country**	**Number of participants (m/f)**	**Age Mean** ± **SD**	**Level/type of activity**	**Objective**	**Results**
Bencke & Zebis (2011) [38] Denmark	24 (12/12)	m: 23.1 ± 3.4 f: 22.7 ± 3.1	Second best division of Danish National Team Handball League	To examine sex differences in neuromuscular pre-activity during a side-cutting manoeuvre	Significantly lower pre-activity of the ST and BF in females than males. But no significant difference for any of the quadriceps muscles
DeMont & Lephart (2004) [39] USA	34 (17/17)	m: 22.5 ± 3.1 f: 20.1 ± 1.4	Recreationally active (aerobic and strength training 3 × per week)	To determine if the level of pre-activation of the gastrocnemius and hamstring muscles during dynamic activity (downhill walking and running) is affected by sex	Significantly higher activity level of the ST in females than males during walking downhill. No sex differences in the other muscles and no differences for any muscle during running
Deschenes et al. (2009) [40] USA	20 (10/10)	m: 21.4 ± 0.8 f: 20.9 ± 0.2	Recreationally active (moderate level)	To examine whether men and women experience different adaptations to muscle unloading (1 week of muscle unloading -walking with a brace and crutches)	No sex differences in the pre-unloading phase. Significantly higher activity in males than in females after one week of unloading. And significant decline in EMG activity from pre- to post-unloading in females
Hanson et al. (2008) [24] USA	40 (20/20)	m: 19.4 ± 1.4 f: 19.8 ± 1.1	NCAA division I level soccer players	To examine sex differences in lower extremity muscle activation between male and female soccer athletes during 2 side-step cutting manoeuvres (running and box-jump side-step cut)	Significantly higher activity in VL in females than males during both preparatory and loading phase of both cutting tasks. Significant increase from preparatory to loading phase for both sexes. Females showed a significantly greater Q:H coactivation ratio than males. No significant differences for the other muscles in both phases and tasks
Hart et al. (2007) [41] USA	16 (8/8)	m: 19.1 ± 1.4 f: 22.0 ± 2.1	Division I soccer players	To evaluate sex differences in muscle activity while landing from a 100 cm single leg forward jump	No sex differences for VL, BF or MG was found
Kim et al. (2016) [42] USA	40 (20/20)	m: 20.45 ± 1.57 f: 20.05 ± 1.23	Physically active (university population)	To assess whether preparatory and reactive knee stiffening strategies are affected differently in males and females exposed to sex-biased cognitive loads	No sex differences were observed
Lee et al. (2014) [43] USA	43 (21/22)	m: 25.0 ± 3.7 f: 24.5 ± 3.6	Recreationally active	To investigate sex differences in pivoting neuromuscular control during strenuous stepping tasks and proprioceptive acuity under weight-bearing	Significantly higher entropy of time-to-peak EMG in the MG during MIPT and MEPT, and in the LG during MIPT in females
Myer et al. (2005) [27] USA	20 (10/10)	m: 25.5 ± 2.7 f: 22.3 ± 3.7	Physically active (students; sport activity at least 1 × per week)	To evaluate sex differences in quadriceps muscle activation strategies when performing a manoeuvre (side-step exercise) that mimics the high ACL injury risk position	No sex differences in normalized VM or VL. But the VM-to-VL ratio was significantly decreased in females compared to males
Padua et al. (2005) [28] USA	21 (11/10)	m: 27.81 ± 4.35 f: 24.10 ± 3.75	Recreational experience in jumping/landings sports (basketball, volleyball, soccer)	To compare leg stiffness, muscle activation, and joint movement patterns between men and women during 2-legged hopping at two different rates	Significantly higher quadriceps activity in females than males in both hopping conditions. The quadriceps activity was significantly higher in the loading phase for both females and males. No significant difference in hamstrings activity. No significant differences between sexes for the gastrocnemius activity; but there was a significant difference between both phases and frequencies. Significantly higher Q:H ratio in females—quadriceps activation was 2 × higher than hamstrings for both hopping conditions
Palmieri-Smith et al. (2007) [44] USA	21 (10/11)	m: 23.6 ± 3.8 f: 24.0 ± 5.2	Recreationally active	To examine the relationship between the peak valgus knee angle and preparatory muscle activity while performing a single-leg forward hop	A higher peak VKA was associated with increased preparatory VL and lateral hamstring activity, while a lower VKA was associated with increased preparatory VM activity in females. When both sexes or males alone were considered, no such results were found
Rozzi et al. (1999) [45] USA	34 (17/17)	m: 20.4 ± 1.7 f: 18.9 ± 0.9	Collegiate basketball and soccer players	To examine knee joint laxity and the neuromuscular characteristics of male and female athletes (task during EMG measurement: a single-leg jump from a step)	Significantly greater peak amplitude in the first contraction after landing in females than males for BF. No significant differences between groups for the other muscles were found
Shultz et al. (2001) [20] USA	64 (32/32)	m: 20.5 ± 1.2 f: 19.4 ± 1.0	Intercollegiate athletes (soccer and lacrosse)	To examine whether muscle response times and activation patterns in the lower extremity differed between men and women in response to a rotational knee perturbation while standing in a single-leg, weight-bearing stance	Females responded significantly faster than males for both ER and IR. This difference appeared to be primarily due to shorter latency in quadriceps activation in females. No significant sex-by-muscle group or muscle-side interactions were found
Smith et al. (2009) [46] USA	26 (12/14)	over both sexes: 24.5 ± 2.7	Recreationally active	To investigate the effects of fatigue and sex on frontal plane knee motion, EMG amplitudes, and GRF magnitudes during a bilateral drop-jump landing	No significant sex differences observed. Also, no differences for fatigue by sex interaction. Only a significant difference between pre-fatigued and fatigued condition
Urabe et al. (2004) [22] Japan	15 (7/8)	m: 22.2 ± 0.4 f: 22.0 ± 1.0	National collegiate basketball league	To determine the activation level of the quadriceps and hamstring muscles during a vertical jump (bilateral) with a single-leg landing	Significantly higher activity of the VM in females at knee flexion angle of 15–45°. No significant differences in overall mean hamstring activity at the same knee flexion range. But when the knee flexion angle was 15°, 20° and 25°, hamstring activity was significantly lower in females
Wu et al. (2016) [47] Ireland	44 (22/22)	m: 23.7 ± 4.2 f: 23.5 ± 3.4	Recreationally active (no strength training or competitive sports within the last 5 years)	To concurrently assess the effect of age on neuromuscular and mechanical properties in 24 young and 20 older males and females	Significantly lower VL activation in older compared to young participants and in females compared to males. BF co-activation was significantly higher in females than males but with no difference between young and old participants. But BF co-activation was similar for both males and females and for young and old after adopting subcutaneous fat as a covariate

Legend: *SD* standardized difference, *m* male, *f* female, *NCAA* National Collegiate Athletic Association, *EMG* electromyography, *ACL* anterior cruciate ligament, *GRF* ground reaction forces, *MIPT* motor internal perturbation task, *MEPT* motor external perturbation task, *VKA* valgus knee angle, *RF* rectus femoris, *VL* vastus lateralis, *VM* vastus medialis, *ST* semitendinosus, *BF* biceps femoris, *MG* medial gastrocnemius, *LG* lateral gastrocnemius, *SM* semimembranosus, *ER* external rotation, *IR* internal rotation

### Synthesis of study results

Three studies found a significantly higher activity in the muscles VL and VM in females, compared to males during a vertical jump, two side-step cutting manoeuvres and two-legged hopping at two different rates [22, 24, 28]. Two studies reported significantly lower neuromuscular activity in the BF and ST in females compared to males during a vertical jump and a side-cutting manoeuvres [22, 38]. No significant differences of neuromuscular activation between females and males, or even contradicting results [39, 47] were found in all other included studies (see Table 4). The effect size of six studies [20, 38, 40, 44, 46, 47] favour female sex, and nine [22, 24, 27, 28, 39, 41–43, 45] favour male sex for higher neuromuscular activation. The effect sizes presented showed a small to medium effect [49]. Only two studies reported a large effect: Deschenes et al. (2009) [40] reported an effect size of -1.71 (95%-CI: -2.77; -0.65), and Wu et al. (2016) [47] an effect size of -0.74 (95%-CI: -1.18; -0.31). Moreover, the calculated effect size of nine studies indicates a p-value higher than 0.5 as the 95%-CI includes the null hypothesis and thus does not favour either sex [22, 27, 38, 39, 41, 42, 44–46].

**Table 4:** Overview of study outcome measurements with values for measured muscles of males (m) in the upper row and for females (f) in the lower row

**Study (publication year)**	**Task**	**Mean ± SD of measured muscles (m/f)**							Upper row: Data normalisation [unit] lower row: Effect size^*^(95%-CI) comparing males to females
		RF	VL	VM	ST	BF	MG	LG	
Bencke & Zebis (2011) [38]	side-cutting maneuver		64 ± 36 53 ± 22	61 ± 25 69 ± 24	46 ± 14 33 ± 12	52 ± 22 30 ± 10			MVC [%] -0.54 (-1.22; 0.13)
DeMont & Lephart (2004) [39]	jogging			31.74 ± 13.40 27.03 ± 13.14	39.71 ± 13.84 43.10 ± 10.22	36.96 ± 18.16 37.84 ± 11.59	38.19 ± 22.30 41.02 ± 22.18	20.69 ± 20.01 21.66 ± 16.88	MVC [%] 0.15 (-0.04; 0.35)
	walking			26.66 ± 12.62 26.68 ± 12.24	23.04 ± 8.60 31.73 ± 9.90	25.06 ± 11.13 28.54 ± 10.10	15.82 ± 17.87 18.27 ± 21.85	7.09 ± 6.02 7.52 ± 5.05	
Deschenes et al. (2009) [40]	pre-unloading		**average of VL & VM** 304.3 ± 44.4 236.8 ± 29.7						RMS raw data [Mv x S] -1.71 (-2.77; -0.65)
Hanson et al. (2008) [24]	running side-step cut preparatory phase	80.19 ± 47.84 80.25 ± 38.46	129.36 ± 63.30 186.14 ± 102.75		77.46 ± 57.63 72.19 ± 34.75	194.92 ± 113.68 172.29 ± 64.43			MVC [%] 0.27 (0.08; 0.46)
	running side-step cut loading phase	136.73 ± 63.46 173.32 ± 81.08	188.85 ± 61.60 320.86 ± 164.65		128.02 ± 48.60 130.22 ± 92.68	194.18 ± 143.23 210.57 ± 85.13			
	box-jump side-step cut preparatory phase	33.04 ± 19.53 40.20 ± 26.41	51.57 ± 22.53 77.15 ± 51.97		32.16 ± 24.80 34.82 ± 20.57	137.35 ± 75.56 122.53 ± 61.7			
	box-jump side-step cut loading phase	107.17 ± 58.06 140.55 ± 75.15	151.94 ± 50.47 244.45 ± 128.24		106.64 ± 50.05 119.37 ± 75.45	154.75 ± 133.71 154.86 ± 69.38			
Hart et al. (2007) [41]	100 cm single-leg forward jump		16.91 ± 21.25 14.51 ± 7.52			5.85 ± 4.44 8.23 ± 5.92	2.79 ± 4.17 2.30 ± 2.33		RMS submax load [mV] 0.05 (-0.52; 0.62)
Kim et al. (2016) [42]	rapid eccentric knee extension	**quadriceps & hamstrings** 42.927 ± 57.167 53.277 ± 57.167							MVC [%] 0.18 (-0.44; 0.8)
Lee et al. (2014) [43]	regular stepping task		2.48 ± 0.52 2.42 ± 0.51	2.34 ± 0.58 2.45 ± 0.58	2.70 ± 0.74 2.73 ± 0.50	2.63 ± 0.42 2.70 ± 0.47	1.95 ± 0.84 2.07 ± 0.72	2.18 ± 0.74 2.43 ± 0.85	time to peak [mV] 0.34 (0.2; 0.49)
	motor internal perturbation task		2.61 ± 0.36 2.74 ± 0.43	2.48 ± 0.58 2.61 ± 0.44	2.71 ± 0.54 3.00 ± 0.45	2.77 ± 0.48 2.89 ± 0.40	2.41 ± 0.64 2.93 ± 0.62	2.60 ± 0.43 2.99 ± 0.52	
	motor external perturbation task		2.84 ± 0.38 2.98 ± 0.40	2.61 ± 0.53 2.82 ± 0.38	2.64 ± 0.43 2.84 ± 0.41	2.86 ± 0.36 2.94 ± 0.44	2.76 ± 0.57 3.18 ± 0.57	2.82 ± 0.57 3.05 ± 0.66	
Myer et al. (2005) [27]	side-step exercise		0.518 ± 0.351 0.689 ± 0.235	0.511 ± 0.207 0.507 ± 0.181					RMS submax load [ratio] 0.26 (-0.37; 0.88)
	side-step exercise		0.577 ± 0.391 0.834 ± 0.366	0.581 ± 0.264 0.596 ± 0.305					MAR submax load [ratio] 0.36 (-0.27; 0.99)
Padua et al. (2005) [28]	pref preparatory phase	16.80 ± 18.66 31.00 ± 20.56			19.00 ± 10.44 23.30 ± 11.28		86.10 ± 67.99 118.20 ± 74.62		MVC [%] 0.41 (0.15; 0.66)
	3.0 preparatory phase	19.60 ± 18.97 39.50 ± 20.89			23.20 ± 14.55 21.90 ± 15.92		104.70 ± 89.81 160.70 ± 98.84		
	pref loading phase	37.70 ± 8.26 67.10 ± 42.12			25.80 ± 10.44 24.00 ± 11.61		135.50 ± 89.81 160.00 ± 98.84		
	3.0 loading phase	34.40 ± 29.09 65.30 ± 32.17			27.30 ± 10.75 22.60 ± 11.61		144.50 ± 71.78 186.60 ± 78.94		
Palmieri-Smith et al. (2007) [44]	single-leg forward jump	1.29 ± 1.38 1.02 ± 1.07	2.43 ± 2.56 2.71 ± 2.87	3.03 ± 3.21 2.49 ± 2.63	1.16 ± 1.16 1.50 ± 1.53	1.95 ± 1.88 1.32 ± 1.19			MVC [%]-0.09 (-0.47; 0.3)
Rozzi et al. (1999) [45]	single-leg jump from a step		298.00 ± 231.27 315.82 ± 162.24	290.87 ± 173.62 361.65 ± 255.49	134.20 ± 66.33 163.49 ± 84.45	84.84 ± 43.47 156.00 ± 72.59	134.13 ± 74.70 225.86 ± 223.35	161.45 ± 73.82 131.72 ± 64.90	first contraction amplitude[mV] 0.33 (-0.07: 0.74)
Shultz et al. (2001) [20]	single-leg external rotation		100.0 ± 24.0 90.8 ± 16.3	99.0 ± 22.8 87.1 ± 18.0	60.5 ± 7.5 59.6 ± 10.6	72.4 ± 17.2 68.4 ± 14.0	56.6 ± 8.0 55.1 ± 8.4	59.2 ± 10.0 56.2 ± 8.3	reaction time [ms] -0.32 (-0.47: -0.18)
	single-leg internal rotation		98.8 ± 22.4 89.8 ± 12.6	95.7 ± 20.4 86.3 ± 13.6	60.5 ± 10.3 56.3 ± 11.6	80.0 ± 25.8 73.8 ± 18.2	54.1 ± 8.8 52.4 ± 9.0	57.0 ± 10.0 55.2 ± 8.8	
Smith et al. (2009) [46]	pre-fatigue		77.1 ± 7.4 73.5 ± 7.7	74.1 ± 9.4 77.5 ± 6.8	70.6 ± 9.8 71.6 ± 12.4	77.3 ± 36.2 70.4 ± 14.0	70.2 ± 7.2 70.8 ± 7.2		RMS submax load [%] -0.03 (-0.37; 0.32)
Urabe et al. (2004) [22]	vertical jump—single-leg landing		158.4 ± 67.0 229.5 ± 108.3	140.0 ± 51.3 216.2 ± 54.0	**SM** 42.6 ± 24.3 42.3 ± 11.7	49.7 ± 7.3 45.3 ± 12.7			MVC [%] 0.39 (-0.36; 1.13)
Wu et al. (2016) [47]	young—leg dynamometer		0.214 ± 0.063 0.160 ± 0.049			18.0 ± 10.2 25.9 ± 9.4			RMS [VL = mV; BF = %] VL: -0.74 (-1.18; -0.31) BF: 0.6 (0.17; 1.03)
	older—leg dynamometer		0.158 ± 0.073 0.119 ± 0.063			16.2 ± 10.7 20.4 ± 8.5			

Legend: ^*^Weighted overall effect size per study; negative = higher activation in female; positive = higher activation in male; *SD* standardized deviation, *RF* rectus femoris, *VL* vastus lateralis, *VM* vastus medialis, *ST* semitendinosus, *BF* biceps femoris, *MG* medial gastrocnemius, *LG* lateral gastrocnemius, *SM* semimembranosus, *%MVC* maximal voluntary contraction, *RMS* root mean square, *MAR* maximum amplitude, *pref* preferred

One study had a fatigue-protocol which was applied after the baseline measurement to examine the effect of fatigue [46]. Another study performed an unloading-protocol by walking on crutches and with a fixed knee brace for one week after baseline measurement [40]. From these two studies, only baseline measurements were included in the results as the aspect of fatigue was part of the exclusion criteria. A follow-up study had to be excluded, because the same data as in the first study was used as baseline data (see Fig. 1).

## Discussion

The aim of this systematic review was to determine whether there are sex differences in neuromuscular activation of knee stabilizing muscles. Only seven studies reported EMG activity as %MVC, with the main findings of a significantly higher activation in VL/VM [22, 24, 28] and significantly lower activation in the hamstrings [22, 38] in females. But one study [39] found significantly higher activation in the hamstrings in females and two studies [42, 44] found no significant sex-difference. However, these studies differed in terms of the task performed during the measurement. Furthermore, the eight studies which had another normalisation of their EMG activity also showed opposing results [20, 27, 40, 41, 43, 45–47].

There are some differences between the registered protocol in PROSPERO and the review: Post-hoc changes to eligibility criteria had to be made and pre-specified inclusion criteria had to be adapted due to insufficient data [50]. Moreover, the risk of bias assessment was evaluated using the QAT from the NHLBI [37] instead of the Downs and Black Quality Index [51]. The QAT is better suited for the quality assessment of cross-sectional studies.

There are many different quality assessment tools for cross-sectional studies, but no gold standard exists [52]. The NHLBI assessment tool QAT [37] was used to reason why some questions were not applicable for cross-sectional studies addressing different tasks with EMG measurements. For instance, assessor blinding was not mentioned in the studies. However, it can be assumed that blinding was given by detection of objective EMG data with the computer. Moreover, the computer analysis could be carried out by a second researcher who had not been involved in the recruitment and measurement process. To overcome this problem, future studies should report the exact data collection procedure. The QAT [37] is widely used and recommended by Ma et al. (2020) [53] and Carbia et al. (2018) [54] for the quality assessment of observational cohort and cross-sectional studies [53, 54]. The evaluation of the overall quality rating remains subjective to a certain extent as no scores are awarded. This subjectivity is somewhat reduced by the QAT guidelines [37]. The guidelines force the researcher to look at the overall picture of the individual studies including their methodological differences as well as how the various aspects of the QAT [37] have to be weighted in reference to the research questions. In addition, the total agreement between the researchers and the inter-rater reliability was calculated which can objectify the results of the quality assessment.

The results from the five studies with poor quality showed contradictory results. A significant higher activation or time to peak in women was found in three studies, but in different muscles [42, 46, 29]. One study found a lower activation in the VL in women [50], and one found no significant difference [49]. The seven studies which had a data normalisation with %MVC still differed in the task carried out during the measurement [23, 25, 29, 41, 42, 45, 47]. The same problem concerned all the studies which were rated having good quality [21, 41, 44, 48]. Even though three studies found sex-specific differences in neuromuscular activation, they all used a different data normalisation method [21, 41, 48]. Therefore, subgroup analyses were also not possible. The methodological limitations in the selected studies, which made the quantitative summarization by a meta-analysis impossible, was also found in previous systematic reviews [55, 56]. According to Martin-Fuentes et al. (2020) [56], the methodological issue is the main concern for the interpretation of EMG data and involves a potential risk of bias. Therefore, it is suggested that future studies should account for these issues.

Several differences in the study characteristics of the included studies were found but this was not the case for the study design as they were all cross-sectional studies. Even though most of the participants were within the same age range between 20 to 30 years and healthy, the results cannot be generalized for this population only due to the uncertainty of the results. The fact that only studies with adult participants were included lead to a small number of eligible hits from the databases. Many studies had to be excluded which had examined the neuromuscular control in adolescents. However, this decision can be justified by the fact that adolescence is a time of physical change and hormonal adaption which could have an influence on sex-specific neuromuscular activation [57].

Five studies also assessed activation of further muscles (gluteus medius and maximus, soleus and tibialis anterior) which are not directly related to knee stability [24, 28, 39, 41, 44]. Therefore, these muscles were not included in this systematic review. Also, all other outcomes in the included studies such as kinematics and joint laxity were not considered in this systematic review.

The level or type of sport varied between the studies and this could have distorted the results as the activity level could have an influence on the sex-specific neuromuscular activation [58]. The performance of the different tasks in the studies depended on the sport level and whether the participants were familiar with the tasks. For example, jogging, jumping one- and two-legged, or side-cutting and -stepping manoeuvres were used to assess neuromuscular activation of participants. In order not to narrow the search too much, the task was not specified in advance. The question arises whether it is appropriate to compare neuromuscular activity between different tasks to evaluate sex-specific neuromuscular activation and not a particular task. The systematic review of Benjaminse et al. (2011) [29] laid their focus on the plant and cut manoeuvres and thereby only had two references which examined the sex-specific neuromuscular activation with surface EMG. Another systematic review limited the tasks to dynamic activities and searched for neuromuscular control in ACL patients but did not compare between sexes [59].

The selection of included EMG data was restricted a priori to variables in the domains of amplitude (mean, RMS, integral) and time (onset, offset, time to peak). Frequency analysis of EMG data was not appropriate for the research question related to dynamic movement situations. Therefore, studies which assessed sex-specific differences under the aspect of fatigue were excluded. In addition, data normalisation varied between the included studies, which did not allow to pool data adequately. This problem had also been observed in previous reviews [55, 56]. EMG data normalization for lower extremity could be done by taking neuromuscular activation during treadmill walking as 100% [60, 61].

The sample size had a large effect on the significance of the results leading to lower power [29]. This fact could be one reason for the heterogenous results. Only four studies provided a sample size justification. Moreover, the sample size was small with a mean of fifteen subjects included in each intervention or control group [38, 42, 43, 47]. The question remains whether a conclusive result can be drawn from a cross-sectional study design with small sample sizes. For a better understanding, cohort studies or randomised controlled trials could show clearer results.

As the results showed different and contradictory findings, the question arises whether it is purposeful to examine isolated neuromuscular activation. Factors such as anatomy, biomechanics and hormones could influence the measurements [62]. Significant differences in foot and hip positions during a single-legged squat in women and men were associated with a decreased ability for women to maintain a varus knee position [63]. Benjaminse et al. (2011) [29] questioned whether the ACL injuries during plant and cutting manoeuvres were sex-related and stated that “the biomechanical and neuromuscular contributions to injury risk should not be isolated and should extend beyond an isolated gender focus”. Furthermore, participants with different body fat levels should be analysed since this variable could influence the EMG activity recorded [64].

Another interesting aspect could be the influence of unloading which could explain the sex difference in increased risk of injury. One of the included studies found out that after one week of unloading men had a significantly higher activity and the decline in EMG activity from pre- to post-unloading was significant in women [40]. The conclusion was “that women suffer a greater degree of neuromuscular disturbance than men as a result of short-term muscle unloading” [40]. These results suggested that women might be more sensitive to a lack of neuromuscular stimulus. One could conclude that this could have a large influence on the rehabilitation after injury and that preventive muscle training could be more important for women than for men. Therefore, future studies assessing patients with an ACL rupture are needed to further understand how the injury itself, treatment modalities, or sex influences neuromuscular activation.

## Conclusion

This systematic review provides an objective overview of the current research available about sex-specific differences in neuromuscular activation of the lower extremity in healthy adults. The controversial findings do not allow for a concluding answer to the question of sex-specific neuromuscular activation of the lower extremity. In addition, the study quality of the included studies was mainly fair, which suggest the need of more high-quality research to draw a solid conclusion and reduce the risk of bias. Specifically, further research with higher statistical power and a more homogeneous methodical procedure (tasks and data normalisation) of the included studies may help to evaluate whether a sex-specific difference in neuromuscular activation exists. Beside from that, conducting studies with ACL-injured subjects and healthy controls would be important to find out how neuromuscular activation may change in women and men after an ACL injury.

## Supplementary Information

**Additional file 1: PRISMA 2020 Checklist.**

## Data Availability

All data generated or analysed during this study are included in this published article.

## Abbreviations

EMG: Electromyography
ACL: Anterior cruciate ligament
PRISMA: Preferred Reporting Items for Systematic Reviews and Meta-Analysis
PROSPERO: Prospective Register of Systematic Reviews Database
PECO: Population/Exposure/Comparator/Outcome
QAT: Quality Assessment Tool for Observational Cohort and Cross-Sectional Studies
NHLBI: National Heart, Lung and Blood Institute
MVC: Maximum voluntary contraction
RMS: Root mean square
RF: Rectus femoris muscle
VL: Vastus lateralis muscle
VM: Vastus medialis muscle
ST: Semitendinosus muscle
BF: Biceps femoris muscle
MG: Medial gastrocnemius muscle
LG: Lateral gastrocnemius muscle
CENTRAL: Cochrane Central Register of Controlled Trials
SM: Semimembranosus muscle
